# RNA-sequencing in non-small cell lung cancer shows gene downregulation of therapeutic targets in tumor tissue compared to non-malignant lung tissue

**DOI:** 10.1186/s13014-018-1075-1

**Published:** 2018-07-17

**Authors:** Kobe Reynders, Els Wauters, Matthieu Moisse, Herbert Decaluwé, Paul De Leyn, Stéphanie Peeters, Maarten Lambrecht, Kristiaan Nackaerts, Christophe Dooms, Wim Janssens, Johan Vansteenkiste, Diether Lambrechts, Dirk De Ruysscher

**Affiliations:** 1 Experimental Radiation Oncology, Department of Oncology, KU Leuven, Leuven, Belgium; 2 Radiation Oncology Department, University Hospitals Gasthuisberg, KU Leuven, Leuven, Belgium; 3 Respiratory Oncology Department, University Hospitals Gasthuisberg, KU Leuven, Leuven, Belgium; 4 Vesalius Research Center (VRC), VIB, KU Leuven, Leuven, Belgium; 5 Laboratory for Translational Genetics, Department of Oncology, KU Leuven, Leuven, Belgium; 6 Department of Thoracic surgery, University Hospital Gasthuisberg, KU Leuven, Leuven, Belgium; 7 Respiratory Division, University Hospital Gasthuisberg, KU Leuven, Leuven, Belgium; 8 Department of Radiation Oncology (Maastro Clinic), Maastricht University Medical Center, GROW, Maastricht, The Netherlands; 9 Lab of Experimental Radiotherapy, UH-Gasthuisberg, CDG-8th floor-box 815, Herestraat 49 – 3000, Leuven, Belgium

**Keywords:** Targeted treatment, NSCLC, PD-L1, RNA-sequencing

## Abstract

**Background:**

Gene expression of specific therapeutic targets in non-malignant lung tissue might play an important role in optimizing targeted therapies. This study aims to identify different expression patterns of fifteen genes important for targeted therapy in non-small cell lung cancer (NSCLC).

**Methods:**

We prospectively collected tissue of NSCLC and non-malignant lung tissue from 25 primary resected patients. RNA-sequencing and 450 K methylation array profiling was applied to both NSCLC and non-malignant lung tissue and data were analyzed for 14 target genes. We analyzed differential expression and methylation as well as expression according to patient characteristics like smoking status, histology, age, chronic obstructive pulmonary disease, C-reactive protein (CRP) and gender. TCGA data served as a validation set.

**Results:**

Nineteen men and 6 women were included. Important targets like *PD-L2* (*p* = 0.035), *VEGFR2* (*p* < 0.001) and *VEGFR3* (*p* < 0.001) were downregulated (respective fold changes = 1.8, 3.1, 2.7, 3.5) in tumor compared to non-malignant lung tissue. The TCGA set confirmed these findings almost universally. *PD-L1* (*p* < 0.001) became also significantly downregulated in the TCGA set. In NSCLC, *MUC1* (*p* = 0.003) showed a higher expression in patients with a CRP < 5 mg/L compared to > 5 mg/L. In the TCGA data but not in our primary data*, PD-L1 & 2* were both borderline more expressed in tumors of active smokers vs. tumors of ex-smokers (*p* = 0.044 and 0.052).

**Conclusions:**

Our results suggest a lower *PD-L1 & 2* and *VEGFR* expression in NSCLC vs. non-malignant lung tissue. Specific patient characteristics did not seem to change the overall expression differences as they were in line with the overall results. This information may contribute to the optimization of targeted treatments.

**Electronic supplementary material:**

The online version of this article (10.1186/s13014-018-1075-1) contains supplementary material, which is available to authorized users.

## Background

Treatment options for metastatic non-small cell lung cancer (NSCLC) are expanding. A lot of effort has been put into therapy options that focus on targeting specific genetic features. The recent addition of immunotherapy to this therapy spectrum has gained a lot of interest because it induces long-lasting remissions in a subset of patients with metastasized NSCLC [1]. However, side effects can occur in varying degrees and their relationship to antitumor efficacy is poorly understood. E.g., pneumonitis is an important, sometimes fatal side effect that requires close attention when using immune checkpoint inhibitors [2]. A recent meta-analysis estimated the rate of pneumonitis (all grades) for programmed death receptor 1 (*PD-1)* and programmed death receptor ligand 1 (*PD-L1)* inhibition at 3.2% [3]. In general, it is not well known which factors influence the occurrence of side effects in targeted NSCLCtherapies and how they relate to therapy efficacy. The expression of a target is used as a predictive and/or prognostic marker for outcome for targeted therapy, but is only semi-quantified through immunohistochemistry on tumor tissue alone [4, 5]. It might be argued that analogous to predictive biomarkers, the differential expression of a gene target in the tumor compared to the non-malignant tissue is related to the toxicity profile of their respective targeted drugs [6].

Methylation patterns are known to influence gene expression in cancer and are more and more used as potential biomarkers for cancer therapy as well [7]. Searching for differential methylation in specific genes might explain their gene expression profile. RNA-sequencing studies mostly focus on whole-exome sequencing.

This study aims to provide insight into the expression differences between NSCLC and non-malignant lung tissue, as to the best of our knowledge, limited matched, comparative RNA-sequencing and methylation data of these targets is available. This data may be useful to assess the tumor specificity of targeted drugs in future clinical research as it can aid in understanding differences in effect/toxicity on surrounding non-malignant lung tissue vs. tumor tissue.

## Methods

### Gene selection

As the major focus of this study, we selected four immune regulatory genes important in targeted therapy. Programmed death receptor 1 *(PD-1*) and both ligands, programmed death receptor ligand 1 & 2 *(PD-L1* and *PD-L2*), were included because of their recent importance in NSCLC treatment [1, 8]. E.g., Pembrolizumab (anti-*PD-1*) is approved in the first-line setting in NSCLC patients with PD-L1 expression > 50% and durvalumab (anti-*PD-L1*) showed a substantial progression free survival benefit when administered as consolidation after chemoradiotherapy [9, 10]. Cytotoxic T-lymphocyte associated protein 4 (*CTLA-4)* was selected because of its potential in several combination treatments that try to lift the brakes on tumor immune response [11]. As for other targets, *EGFR* has already proven its place in adenocarcinoma [12]. The fusion gene of echinoderm microtubule-associated protein-like 4 and anaplastic lymphoma kinase *EML4-ALK* was not included as this does not occur in non-malignant lung tissue. Vascular endothelial growth factor receptor (*VEGFR)* 1, 2 and 3 were looked at because of the importance of blood vessel formation in tumor growth, which has been a target in NSCLC [13]. High expression of hepatocyte growth factor receptor (*MET)* and its ligand hepatocyte growth factor (*HGF)* was correlated with a worse outcome and a higher resistance to *EGFR*-inhibitors in NSCLC [14]. Therefore, *MET* has been suggested as a prognostic and therapeutic target in NSCLC and was included in this study. Similarly, cell surface associated Mucin 1 (*MUC1)* also proved multiple times that it is associated with a poor outcome and that silencing it may lead to reduced tumor survival [15]. Lastly, we included two targets that fell out of the picture in recent years but might play a role when additional research becomes available. The receptor tyrosine kinase gene *KIT* had initially promising preclinical results, but has not yet been backed up with clinical success [16]. Human epidermal growth factor receptor 2 (*HER2)* as a clinical research target has slowed down as well after the first trials showed poor results [17]. Melanoma-associated antigen 3 (*MAGEA3)*, a cancer-testis gene that is consequentially not expressed in non-malignant lung tissue, was included as an internal control.

### Patient inclusion

This prospective dataset and its initial methodology were developed by Wauters et al. [18]. Patients that were eligible for primary, curative-intent surgery for NSCLC were included. From the lobectomy specimen, a fresh frozen biopsy was taken of tumor and non-malignant lung tissue, the latter as distant as possible from the tumor location. Biopsies were gathered and processed immediately after surgery as described in [18]. Clinical and lung function data were obtained from the patient’s medical files. Patients were divided into clinical subsets according to relevant clinical criteria for non-small cell lung cancer. Smoking status was defined as active smoker on the day of surgery or ex-smoker when the patient quit smoking more than 4 weeks before surgery. This 4 week limit is also used by studies that searched for pulmonary, respiratory and wound complications in regards to preoperative smoking cessation [19]. Because there was only one never-smoker, we classified this patient as an ex-smoker for analysis purposes. C-reactive protein (CRP) subsets were determined by a CRP-level higher than 5 mg/L or not. This threshold is routinely used in clinical practice where a CRP > 5 mg/L is associated with an increased inflammatory state. Furthermore, we sub-grouped patients according to gender, histology and age. An age cut-off of 70 years was used, as this is regularly used to define an elderly population in non-small cell lung cancer [20]. Chronic obstructive pulmonary disease (COPD) status - determined by the FEV1/FVC ratio (forced expiratory volume per second / forced vital capacity) was determined as well.

### Validation set

From the Genomic Data Commons Data Portal, we collected all patient entries that had RNA-sequencing data available from NSCLC and/or non-malignant lung tissue. Clinical data and normalized fragment-per-million-reads gene expression of the fourteen selected genes were drawn from the database. Active smoking is defined as a patient that had a registered smoking history, but no registered year of smoking cessation or stopped smoking in the year of pathologic diagnosis. We censored patients without any available smoking history for this subset analysis. No patient data was available for CRP status in the validation set.

### RNA-sequencing and methylation

Biopsies were assessed by means of histological examination to confirm the malignant status (> 70% malignant epithelial cells) or normal lung histology. RNA extraction of non-malignant lung and tumor tissue was performed and sequenced on an Illumina HiSeq 2000. Illumina sequencing libraries were prepared according to the TruSeq RNA Sample Preparation Guide following the manufacturer’s instructions. Libraries were sequenced using 1 × 58 bp single-end reads, with two indexed samples per lane, yielding about 32.5 million reads per sample. After alignment of the sequencing reads to the human genome, counts for each gene were computed for each sample by use of the HTSeq software (version v0.5.3p3). Bisulfite-converted DNA was hybridized on 450 K BeadChips (Illumina), using the Infinium HD methylation protocol. After controlling for batch effect and normalizing raw intensity data, the methylation level of each interrogated CpG, scored as a β value between 0 and 1, was calculated according to the fluorescent intensity ratio using IMA R-package (v2.1.1) and the SWAN method implemented in the minfi R-package (v1.2.0) [21]. Extensive metholodogy regarding the RNA-sequencing and methylation can be found in the supplemental data of the manuscript of dr. Wauters et al. [18].

### Analyses

Aside from the primary endpoint of tumor vs. non-malignant lung tissue, gene expression was also compared between patient subsets on tumor tissue only and non-malignant lung tissue only as a secondary endpoint. In our matched dataset, matched differential expression analysis was performed using a DESeq protocol after normalization with the EDASeq R package. A Benjamini-Hochberg correction for multiple testing (false discovery rate < 0.05) was applied to designate significantly upregulated or downregulated genes. Because of a small number of target genes and thus no need for prioritization, no threshold for fold change of expression was used as fold change is highly dependent on baseline gene expression by nature. In these differentially expressed genes, differential methylation, starting from normalized β-values and reworked to M-values (M-value = log2(β /(1- β))), was assessed between tumor and non-malignant lung tissue. M-values were used because of their increased statistical validity over β-values when determining differential methylation levels on a limited number of samples [22]. CpG sites were differentially methylated when demonstrating a FDR-adjusted *p* value < 0,05 (Benjamini-Hochberg correction) and an absolute M-difference greater than 0.4 [22]. We used a linear model for differential methylation assessment (Limma R-package). Methylation sites that were located in the gene body or three prime untranslated region according to the 450 K BeadChips annotation by Illumina were not included in the descriptive analysis. Next, the location within a particular gene of each CpG site was identified in the University of Californa, Santa Cruz genome browser (build hg19) and correlated with the presence of a CpG island and/or histone markers H3K4Me1, H3K4Me3 and H3K27Ac. These histone markers indicate regulatory elements for gene expression [23]. Results are expressed as mean ± standard deviation (range).

In our validation set, we performed descriptive statistics and log transformed the RNA-sequencing results because of a skewed distribution. We then used a student t-test to compare expression results and calculated fold changes with the average expression per patient subset. A *p*-value < 0.05 was considered significant.

### Ethics

The local ethical committee approved the study. Written informed consent was obtained from all patients.

## Results

In our primary data set, twenty-five patients with NSCLC were included. Demographic details of the study population are depicted in Table 1. Mean age was 65.8 ± 9.2 years (41–79). Ten patients were current smokers, 14 ex-smokers (stopped smoking more than 4 weeks before surgery) and 1 never-smoker. The never-smoker was a 61 year old male with a FEV1/FVC ratio of 94% and only pantoprazole and L-thyroxine as medication. The mean amount of overall pack years was 36.4 ± 20.79 (0–100). The mean CRP level was 12.79 ± 17.06 mg/L (0.2–54.7 mg/L). The mean FEV1/FVC was 67.24 ± 12% (42–94%). Two patients had papillary, 5 well differentiated, 2 moderately differentiated and 5 poorly differentiated adenocarcinoma. One patient had well differentiated, 5 moderately differentiated and 4 poorly differentiated squamous cell carcinoma. The differentiation of one squamous cell carcinoma was unknown. One patient had a single lung transplant, three years before lobectomy of the tumor in the contralateral, non-transplanted lung. Three patients were on low-dose, long-term oral corticosteroids. One patient underwent surgery after induction chemotherapy.

**Table 1:** Study population

Demographics	N	N
Cases	Experiment	Validation
	25	1122
Gender		
▪ Men	19	670
▪ Women	6	452
Smoking status		
▪ Active smokers	10	164
▪ Ex-smokers	14	437
▪ Never-smoker	1	N/A
Histology		
▪ Non-malignant lung	25	108
▪ Squamous ca.	11	501
▪ Adenoca.	14	513
COPD status		
▪ FEV_1_ / FEV < 70%	15	74
▪ FEV_1_ / FEV > 70%	10	146
CRP level		
▪ CRP < 5 mg/L	14	N/A
▪ CRP > 5 mg/L	11	N/A
Disease stage (TNM 7)		
▪ Stage I	15	575
▪ Stage II	6	322
▪ Stage III	3	189
▪ Stage IV	1	36

Characteristics of the study population. Ex-smoking was defined as a total smoking cessation for at least four weeks before the biopsy

*PD-L2* expression was higher in the non-malignant lung tissue with an overall fold change of 1.87 (*p* = 0.035). *PD-L2* also had a fold-change of 2.69 in favor of non-malignant lung tissue in the subgroup of active smokers (*p* = 0.038). Likewise, *PD-L1* was more expressed in non-malignant lung than in tumor tissue in active smokers, showing a fold change of 5.38 (*p* = 0.013). *VEGFR 3* was more expressed in non-malignant lung tissue in almost all subsets except in active smokers and in patients older than 70 years, respectively. *VEGFR 1* only shows higher expression in non-malignant lung tissue of squamous carcinoma patients (*p* = 0.028). *VEGFR2* is significantly higher expressed in non-malignant lung tissue in all patient subsets except for adenocarcinoma, CRP ≤ 5 mg/L, no COPD and women. There is no single gene that was significantly more expressed in tumor tissue, with the exception of the cancer testis gene *MAGEA3*.

When comparing gene expression between different patient subsets in non-malignant lung tissue alone, no differential expression could be found. Within tumor tissue alone, *MUC1* and *KIT* exhibited increased expression in patients with a low CRP compared to those with a CRP ≥5 mg/L. The results of these analyses can be found in Additional file 1: Table S1 and Additional file 2: Table S2.

Our validation set consists of 1122 patient, the demographics of which are represented in Table 1. Patient database entries originated from February 2010 to November 2014, with pathological diagnoses between 1991 and 2013. The average age was 66.8 ± 9.4 years old (33–90). The mean FEV1/FVC was 77.47 ± 19.32% (2–126%). *P*-values of the expression data of non-malignant lung tissue vs. tumor are depicted in Table 2. Fold changes of each gene, which represents the ratio of the normalized expression of a target in non-malignant lung tissue over the normalized expression in tumor tissue, can be found in Table 3.

**Table 2:**
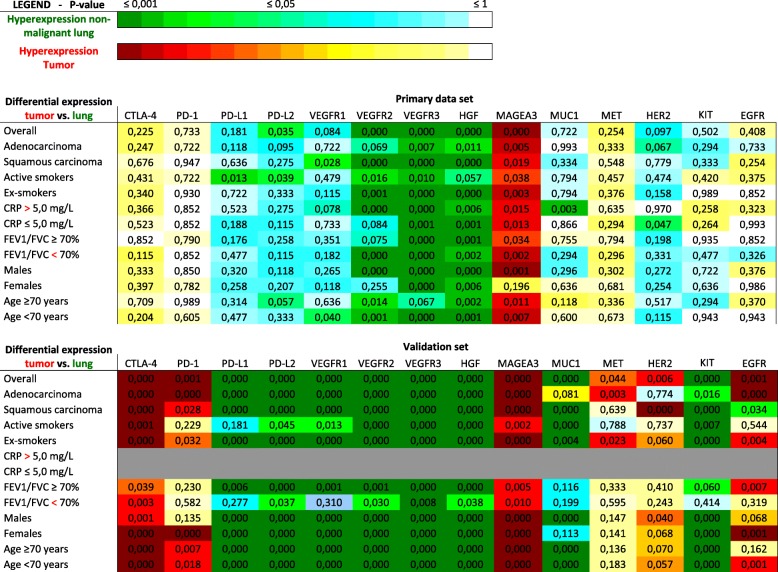
RNA-sequencing results for non-malignant lung tissue vs. tumor tissue – *P*-values

Differential expression of particular genes (top row) in different patient subsets (left column) is represented by *p*-values and color coded. Blue and green colors indicate more expression of a particular gene for a specific patient subset in non-malignant lung tissue compared to tumor tissue. Yellow and red indicate more expression of a particular gene for a specific patient subset in tumor tissue compared to non-malignant lung tissue

**Table 3:**
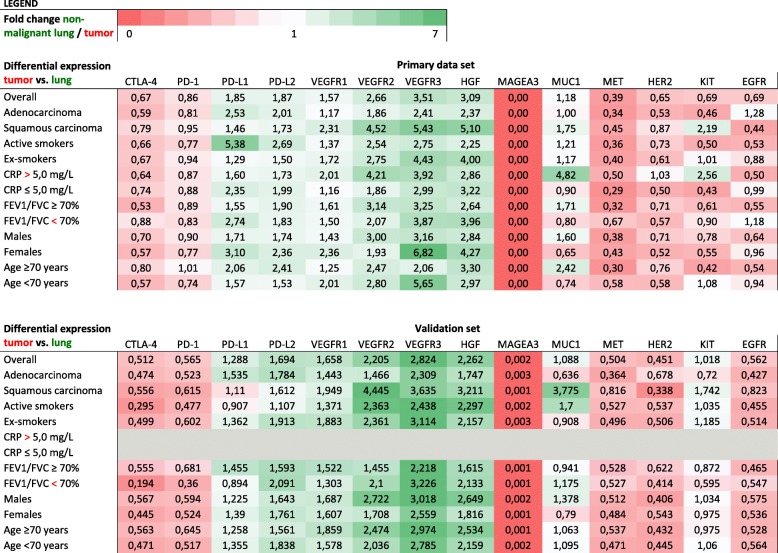
RNA-sequencing results for non-malignant lung tissue vs. tumor tissue – fold changes

Differential expression of particular genes (top row) between different patient subsets (left column) is represented by fold changes and color coded. Fold change is defined as the ratio of the normalized expression of a particular gene for non-malignant lung tissue compared to the expression of that gene in tumor tissue. Green colors indicate a fold change > 1, red indicates a fold change < 1

Methylation results of the differentially expressed genes of our primary data set are represented in Additional file 3: Figure S1. *PD-L1* and *PD-L2* had no significant differential methylation pattern between the tumor and the non-malignant lung tissue. For *PD-L2*, one hypermethylated neoplastic CPG site was found in the lung tissue vs. tumor subset with a corrected *p*-value of 0.006, but an absolute M difference of 0.38 under the preset cut-off (0.4). In tumor tissue, *VEGFR1* has hypermethylated sites in all differentially expressed subsets. *VEGFR2* shows hypermethylation sites in tumor tissue of all differentially expressed subsets except ex-smokers. *VEGFR3* was hypermethylated in tumor tissue in 6 out of 13 subsets.

Hypermethylation in lung tissue was found for *VEGFR1* in the < 70 years subset and for *VEGFR3* in the ex-smokers subset. 80.8% of all CpG sites that are significantly hypermethylated in the tumor are associated with at least 1 histone marker or CpG island. Similarly, of all CpG sites that are significantly hypermethylated in non-malignant lung tissue, 28.6% have this association.

No methylation analysis could be done on the validation data due to absence of minimal TCGA methylation data in both tumor and non-malignant lung tissue.

## Discussion

Most biomarkers for targeted treatments are based upon immunohistochemistry. However, RNA sequencing is proving its value in replacing immunohistochemistry as an accurate method to identify predictive biomarkers [24]. It is to be expected that RNA-sequencing will only gain more importance in patient selection, both for predicting therapy efficacy as well as toxicity [25]. When looking at an individual patient, understanding his/her baseline gene expression of non-malignant lung tissue may provide valuable context for efficacy and toxicity profiles of targeted therapy. With this study, we tried to offer insight into how non-malignant lung and tumor gene expression relate to each other. We therefore analyzed the expression patterns in selected genes for targeted (immune) therapy in twenty-five paired non-malignant lung and NSCLC specimens. The differential expression of these selected genes between matched tumor and the surrounding non-malignant lung tissue is to the best of our knowledge, not known for NSCLC. It is surprising to see that, with some notable exceptions, most target genes in tumor tissue do not have an increase in gene expression when compared to the non-malignant lung tissue, but a decrease. In our patient group, we found that the expression of *PD-L2*, *VEGFR2* and *VEGFR3* was lower in tumor tissue than in the non-malignant lung tissue. Our TCGA data confirmed these findings convincingly. A high tumor expression and a low non-malignant lung tissue expression would seem to be an ideal patient situation for maximum therapy efficiency with a low impact on normal, surrounding lung tissue. However, this does not seem to be the case. For example, *VEGFR 2 & 3* are found on tumor vasculature and both contribute additionally towards angiogenesis [26]. Although we still observed tumor *VEGFR* expression, the 2-3× fold change in favor of non-malignant lung tissue suggests a narrow therapeutic ratio for anti-*VEGFR2* and drugs like bevacizumab. It may be argued that dose limiting toxicities are one of the reasons for the marginal effect of an anti-angiogenesis drug such as bevacizumab [27]. The TCGA data converted the non-significant, overall gene expression in our primary data set into strongly significant, differential expression, due to a vast increase in patient numbers. In the TCGA data, genes like CTLA-4 and PD-1 show an overall increase in tumor gene expression, which did not reach significance in our own patient data. Looking at patient subsets in our primary data set in conjunction with our validation set, it seems that factors like smoking status, age, COPD, histology and CRP level did not impact the overall expression differences we found earlier. In current smokers, *PD-L1* and *PD-L2* had a higher expression in non-malignant lung tissue than in tumor tissue in our primary data set. This can be explained by the inflammatory state of lung parenchyma in active smokers that might increase macrophage count and thus *PD-L1* and/or *PD-L2* expression [28]. Unfortunately, in the validation set, *PD-L1* of tumor vs. non-malignant lung tissue in active smokers is not differentially expressed and ex-smokers do show differential expression. This contradiction might be due to a lack of detailed information about smoking cessation in the Genomic Data Commons Data Portal, which can lead to a false categorization of active/ex-smokers and a number of patients with a complete smoking status that is significantly lower than the total amount of patients available. Secondly, no matched data (tumor and non-malignant lung tissue from the same patient at the same time point) was available in the validation set. Thirdly, non-malignant tissue entries were relatively low. This uncertainty in our validation data and the contradicting results between primary data set and validation set, make us believe that smoking status should still be taken into account in future trials with *PD-1/PD-L1* therapy, not only for response but especially for toxicity.

A reduced gene expression is strongly associated with methylation of CpG sites in gene promotors or enhancers. It is generally hypothesized that cancer cells use this mechanism to silence a wide array of genes that harm their survival [29]. To verify the results of the expression analysis, we analyzed the methylation patterns of all differentially expressed genes in tumor vs. non-malignant lung tissue and looked at their impact of methylation on gene expression. As the impact of a specific methylation site on expression is unknown, we looked at histone markers associated with transcription regulation and found that a high percentage of these sites are located in gene regions that have an impact on expression (enhancers, promoters). Although the interpretation of individually methylated genes is not clear-cut, there are some sound observations to be made here. Of all differentially methylated genes, 67.9% has a methylation profile that supports its expression (meaning more methylated sites on tumor tissue which are correlated with histone markers and/or CpG islands). Only 7.1% of differentially methylated genes show a methylation profile that contradicts their expression. 10.5% of differentially methylated genes do not show any correlation with histone markers but do have more methylated sites on tumor tissue and 10.5% have more methylated sites on non-malignant lung tissue. These changes in methylation patterns are most prominent on the *VEGFR*-genes but are not apparent on the *PD-1, PD-L1* and *PD-L2* family. Overall, the methylation assay hints that most of the differences in gene expression are driven by alterations in the gene methylation profile with the *PD-1*-axis as a notable exception.

The inflammatory status of tissue also plays an important role in the expression results. Different infectious agents might modulate *PD-L1* and CD8+ T cell activity [30]. In a patient population where COPD often is a major factor, the expression of PD-L1 can theoretically shift depending on the current inflammatory status of the lung [31]. In the TCGA validation set, the COPD status was often not known. Of those that had a well-documented COPD status, the majority did not have a FEV1 / FEV < 70%. In our patient data, we did not find major differences between patients with or without COPD, but this might be due to the small patient number. These results do not warrant leaving the COPD status out of the equation in future clinical work.

As the study is explorative in its design, there are some additional caveats to be aware of. The patient population is small. Although we still find convincing differences in expression when comparing non-malignant lung tissue with tumor tissue, this is not the case when comparing tumor with tumor tissue, or non-malignant lung tissue with non-malignant lung tissue. The differences between more similar tissues are allegedly smaller, which makes it difficult to achieve enough statistical power to detect them. Our validation data did largely make up for this by confirming almost all results and detecting even stronger correlations. Another important note is the fact that assessing RNA levels is an indirect measure of expression, and there are many post-translational processes that might affect protein levels and location. Immunohistochemistry is a measure of the end-result of gene expression. However, immunohistochemistry does not allow for a true quantifiable comparison of protein expression and is prone to observer bias.

It is also important to note that this data is obtained from untreated patients. Exposure to chemo- and/or radiation therapy has showed to change expression of certain targets like *PD-L1* [32]. However, targeted treatments like a *PD-1* inhibitor have been approved for first line treatments of advanced disease, making untreated patient data more relevant than before.

Lastly, a further limitation of this study is that a direct link between expression differences and toxicity profile is not within its scope. As this would require a prospective, specific toxicity assessment for each drug individually, it was not achievable with our current study setup.

Our results do provide an interesting hypothesis for further clinical research. The ratio or fold change between the expression of non-malignant lung tissue and tumor tissue might be a patient specific predictor for increased toxicity. An expression analysis of not only tumor tissue, but also non-malignant lung tissue could be implemented in a dedicated clinical trial to see if expression profiles can be predictive for side effects. Currently, this would be especially applicable for the *PD-1/PD-L1* inhibitors, as different levels of *PD-L1* expression are already used as a cut-off for therapy. Expanding the *PD-L1* biomarker to include non-malignant lung tissue and correlating the immunohistochemistry with RNA-sequencing results is an realistic objective that may be part of future prospective trials.

## Conclusion

In this study, we looked for expression differences between non-malignant lung tissue and a non-small cell lung tumor of the same patient. Surprisingly, we found that the expression of most major target genes decreases in tumor tissue compared to the surrounding non-malignant lung. The differential expression of these gene targets might be useful in understanding differences in effects of targeted treatments on tumor and non-malignant lung tissue.

## Additional files

Additional file 1: **Table S1.** RNA-sequencing results for tumor tissue (a) or non-malignant lung tissue (b). Differential expression of particular genes (top row) between different patient subsets (left column) is represented by *p*-values and color coded. Blue and green colors indicate more expression of a particular gene for the first patient subset (red) compared to the second subset (yellow and red). (DOCX 21 kb)

Additional file 2: **Table S2.** Fold changes for tumor tissue (a) or non-malignant lung tissue (b). Differential expression of particular genes (top row) between different patient subsets (left column) is represented by p-values and color coded. Green colors indicate fold change > 1 in favor of the first subset of a particular gene compared to the second subset (red). (DOCX 22 kb)

Additional file 3: **Figure S1.** Differential methylated sites. Representation of the amount of methylated sites per gene and per patient subset. The amount of hypermethylated sites per gene can be found on the y-axis, both for hypermethylated sites in non-malignant lung tissue (blue) and hypermethylated sites in tumor tissue (red). Associations with histone markers and CpG islands are color coded per methylation site. *P*-values can be equal across different subsets and genes for non-significant results because of the statistical method used. (XLSM 509 kb)

## Abbreviations

COPD: Chronic obstructive pulmonary disease
CRP: C-reactive protein
CTLA-4: Cytotoxic T-lymphocyte associated protein 4
EGFR: Epidermal growth factor receptor
EML4-ALK: Echinoderm microtubule-associated protein-like 4 / anaplastic lymphoma kinase
FEV1: Forced expiratory volume per second
FVC: Forced vital capacity
HER2: Human epidermal growth factor receptor 2
HGF: Hepatocyte growth factor
KIT: Mast/stem cell growth factor receptor
MAGEA3: Melanoma-associated antigen 3
MET: Hepatocyte growth factor receptor
MUC 1: Mucin 1
NSCLC: Non-small cell lung cancer
PD-1: Programmed death receptor 1
PD-L1/2: Programmed death receptor ligand 1/2
RNA: Ribonucleic Acid
TCGA: The cancer genome atlas
VEGFR: Vascular endothelial growth factor receptor
