# Supplementary material for: RNA-sequencing in non-small cell lung cancer shows gene downregulation of therapeutic targets in tumor tissue compared to non-malignant lung tissue
Source: Radiat Oncol. 2018 Jul 17;13:131. doi: 10.1186/s13014-018-1075-1 (PMC6050654; doi:10.1186/s13014-018-1075-1)
Supplement: Supplementary file 1 — Table S1. RNA-sequencing results for tumor tissue (a) or non-malignant lung tissue (b). Differential expression of particular genes (top row) between different patient subsets (left column) is represented by p-values and color coded. Blue and green colors indicate more expression of a particular gene for the first patient subset (red) compared to the second subset (yellow and red). (DOCX 21 kb) [file 13014_2018_1075_MOESM1_ESM.docx]

|  |  |  |  |  |  |  |  |  |  |  |  |  |  |  |  |  |  |  |  |  |  |  |  |  |  |  |  |  |  |  |
| --- | --- | --- | --- | --- | --- | --- | --- | --- | --- | --- | --- | --- | --- | --- | --- | --- | --- | --- | --- | --- | --- | --- | --- | --- | --- | --- | --- | --- | --- | --- |
|  | **LEGEND** |  |  |  |  |  |  |  |  |  |  |  |  |  |  |  |  |  |  |  |  |  |  |  |  |  |  |  |  |  |
|  | **Hyperexpression 1st subset** |  |  |  |  |  |  |  |  |  |  |  |  |  |  |  |  |  |  |  |  |  |  |  |  |  |  |  |  |  |
|  | **P-value** | ≤ 0,001 | |  |  |  |  | ≤ 0,05 | |  |  |  |  |  |  |  | ≤ 1 |  |  |  |  |  |  |  |  |  |  |  |  |  |
|  | **Hyperexpression 2nd subset** |  |  |  |  |  |  |  |  |  |  |  |  |  |  |  |  |  |  |  |  |  |  |  |  |  |  |  |  |  |
|  |  |  |  |  |  |  |  |  |  |  |  |  |  |  |  |  |  |  |  |  |  |  |  |  |  |  |  |  |  |  |
|  | **Table 1a** |  |  |  |  |  |  | **Primary data set** | | | | | | | | | | | | | |  |  |  |  |  |  |  |  |  |
|  | **Differential expression tumor** | CTLA-4 | | PD-1 | | PD-L1 | | PD-L2 | | VEGFR1 | | VEGFR2 | | VEGFR3 | | HGF | | MAGEA3 | | MUC1 | | MET | | HER2 | | KIT | | EGFR | |  |
|  | Adenous vs. Squamous | 0,942 | | 0,942 | | 0,942 | | 0,942 | | 0,916 | | 0,916 | | 0,515 | | 0,942 | | 0,942 | | 0,942 | | 0,942 | | 0,942 | | 0,942 | | 0,942 | |  |
|  | Ex-smoking vs. Active smoking | 0,942 | | 0,942 | | 0,169 | | 0,916 | | 0,942 | | 0,942 | | 0,398 | | 0,515 | | 0,942 | | 0,942 | | 0,942 | | 0,935 | | 0,515 | | 0,862 | |  |
|  | CRP low vs. CRP high | 0,942 | | 0,942 | | 0,515 | | 0,942 | | 0,942 | | 0,481 | | 0,942 | | 0,942 | | 0,942 | | 0,004 | | 0,942 | | 0,398 | | 0,132 | | 0,515 | |  |
|  | no COPD vs. COPD | 0,640 | | 0,942 | | 0,942 | | 0,942 | | 0,942 | | 0,942 | | 0,942 | | 0,942 | | 0,935 | | 0,942 | | 0,942 | | 0,942 | | 0,942 | | 0,942 | |  |
|  | Male vs. Female | 0,942 | | 0,942 | | 0,585 | | 0,942 | | 0,515 | | 0,862 | | 0,132 | | 0,916 | | 0,942 | | 0,398 | | 0,942 | | 0,942 | | 0,862 | | 0,942 | |  |
|  | <70 years vs. ≥70 years | 0,942 | | 0,942 | | 0,862 | | 0,942 | | 0,476 | | 0,942 | | 0,398 | | 0,862 | | 0,942 | | 0,515 | | 0,756 | | 0,942 | | 0,916 | | 0,515 | |  |
|  |  |  | |  | |  | |  | |  | |  | |  | |  | |  | |  | |  | |  | |  | |  | |  |
|  | **Table 1b** |  | |  | |  | | **Primary data set** | | | | | | | | | | | | | |  | |  | |  | |  | |  |
|  | **Differential expression lung** | CTLA-4 | | PD-1 | | PD-L1 | | PD-L2 | | VEGFR1 | | VEGFR2 | | VEGFR3 | | HGF | | MAGEA3 | | MUC1 | | MET | | HER2 | | KIT | | EGFR | |  |
|  | Adenous vs. Squamous | 0,985 | | 0,985 | | 0,985 | | 0,985 | | 0,985 | | 0,985 | | 0,985 | | 0,985 | | 0,985 | | 0,985 | | 0,985 | | 0,985 | | 0,985 | | 0,985 | |  |
|  | Ex-smoking vs. Active smoking | 0,985 | | 0,985 | | 0,985 | | 0,985 | | 0,985 | | 0,985 | | 0,985 | | 0,985 | | 0,985 | | 0,985 | | 0,985 | | 0,985 | | 0,985 | | 0,985 | |  |
|  | CRP low vs. CRP high | 0,985 | | 0,985 | | 0,985 | | 0,985 | | 0,985 | | 0,985 | | 0,985 | | 0,985 | | 0,985 | | 0,985 | | 0,985 | | 0,985 | | 0,985 | | 0,985 | |  |
|  | no COPD vs. COPD | 0,985 | | 0,985 | | 0,985 | | 0,985 | | 0,985 | | 0,985 | | 0,985 | | 0,985 | | 0,985 | | 0,985 | | 0,985 | | 0,985 | | 0,985 | | 0,985 | |  |
|  | Male vs. Female | 0,985 | | 0,985 | | 0,985 | | 0,985 | | 0,985 | | 0,985 | | 0,985 | | 1,000 | | 0,985 | | 1,000 | | 0,985 | | 0,985 | | 0,985 | | 0,985 | |  |
|  | <70 years vs. ≥70 years | 0,985 | | 0,985 | | 0,985 | | 0,985 | | 0,985 | | 0,985 | | 0,985 | | 0,985 | | 0,985 | | 0,985 | | 0,985 | | 0,985 | | 0,985 | | 0,985 | |  |
|  |  |  |  |  |  |  |  |  |  |  | |  |  |  | |  | |  |  |  |  |  |  |  |  |  |  |  |  |  |

|  |  |  |  |  |  |  |  |  |  |  | |  |  |  | |  | |  |  |  |  |  |  |  |  |  |  |  |  |  |
| --- | --- | --- | --- | --- | --- | --- | --- | --- | --- | --- | --- | --- | --- | --- | --- | --- | --- | --- | --- | --- | --- | --- | --- | --- | --- | --- | --- | --- | --- | --- |
|  | **Table 1c** |  |  |  |  |  |  | **Validation set** | | | | | | | | | | | | | |  |  |  |  |  |  |  |  |  |
|  | **Differential expression tumor** | CTLA-4 | | PD-1 | | PD-L1 | | PD-L2 | | VEGFR1 | | VEGFR2 | | VEGFR3 | | HGF | | MAGEA3 | | MUC1 | | MET | | HER2 | | KIT | | EGFR | |  |
|  | Adenous vs. Squamous | 0,000 | | 0,001 | | 0,004 | | 0,417 | | 0,000 | | 0,000 | | 0,000 | | 0,000 | | 0,000 | | 0,000 | | 0,000 | | 0,000 | | 0,000 | | 0,000 | |  |
|  | Ex-smoking vs. Active smoking | 0,011 | | 0,044 | | 0,052 | | 0,076 | | 0,417 | | 0,071 | | 0,915 | | 0,400 | | 0,280 | | 0,008 | | 0,092 | | 0,068 | | 0,632 | | 0,132 | |  |
|  | CRP low vs. CRP high |  | |  | |  | |  | |  | |  | |  | |  | |  | |  | |  | |  | |  | |  | |  |
|  | no COPD vs. COPD | 0,006 | | 0,025 | | 0,811 | | 0,030 | | 0,002 | | 0,000 | | 0,000 | | 0,000 | | 0,262 | | 0,002 | | 0,133 | | 0,642 | | 0,054 | | 0,003 | |  |
|  | Male vs. Female | 0,000 | | 0,000 | | 0,621 | | 0,084 | | 0,019 | | 0,000 | | 0,000 | | 0,000 | | 0,002 | | 0,000 | | 0,046 | | 0,742 | | 0,000 | | 0,000 | |  |
|  | <70 years vs. ≥70 years | 0,093 | | 0,195 | | 0,328 | | 0,014 | | 0,382 | | 0,328 | | 0,825 | | 0,110 | | 0,260 | | 0,868 | | 0,675 | | 0,034 | | 0,653 | | 0,763 | |  |
|  |  |  | |  | |  | |  | |  | |  | |  | |  | |  | |  | |  | |  | |  | |  | |  |
|  | **Table 1d** |  | |  | |  | | **Validation set** | | | | | | | | | | | | | |  | |  | |  | |  | |  |
|  | **Differential expression lung** | CTLA-4 | | PD-1 | | PD-L1 | | PD-L2 | | VEGFR1 | | VEGFR2 | | VEGFR3 | | HGF | | MAGEA3 | | MUC1 | | MET | | HER2 | | KIT | | EGFR | |  |
|  | Adenous vs. Squamous |  | |  | |  | |  | |  | |  | |  | |  | |  | |  | |  | |  | |  | |  | |  |
|  | Ex-smoking vs. Active smoking | 0,634 | | 0,387 | | 0,224 | | 0,015 | | 0,066 | | 0,224 | | 0,246 | | 0,496 | | 0,767 | | 0,000 | | 0,016 | | 0,978 | | 0,666 | | 0,379 | |  |
|  | CRP low vs. CRP high |  | |  | |  | |  | |  | |  | |  | |  | |  | |  | |  | |  | |  | |  | |  |
|  | no COPD vs. COPD | 0,003 | | 0,322 | | 0,842 | | 0,410 | | 0,122 | | 0,568 | | 0,318 | | 0,401 | | 0,924 | | 0,540 | | 0,277 | | 0,012 | | 0,224 | | 0,123 | |  |
|  | Male vs. Female | 0,333 | | 0,651 | | 0,907 | | 0,298 | | 0,914 | | 0,439 | | 0,691 | | 0,221 | | 0,928 | | 0,022 | | 0,255 | | 0,789 | | 0,130 | | 0,369 | |  |
|  | <70 years vs. ≥70 years | 0,121 | | 0,983 | | 0,510 | | 0,138 | | 0,261 | | 0,146 | | 0,600 | | 0,930 | | 0,809 | | 0,771 | | 0,964 | | 0,070 | | 0,026 | | 0,004 | |  |
|  |  |  |  |  |  |  |  |  |  |  |  |  |  |  |  |  |  |  |  |  |  |  |  |  |  |  |  |  |  |  |
