# Supplementary material for: RNA-sequencing in non-small cell lung cancer shows gene downregulation of therapeutic targets in tumor tissue compared to non-malignant lung tissue
Source: Radiat Oncol. 2018 Jul 17;13:131. doi: 10.1186/s13014-018-1075-1 (PMC6050654; doi:10.1186/s13014-018-1075-1)
Supplement: Supplementary file 2 — Table S2. Fold changes for tumor tissue (a) or non-malignant lung tissue (b). Differential expression of particular genes (top row) between different patient subsets (left column) is represented by p-values and color coded. Green colors indicate fold change > 1 in favor of the first subset of a particular gene compared to the second subset (red). (DOCX 22 kb) [file 13014_2018_1075_MOESM2_ESM.docx]

|  |  |  |  |  |  |  |  |  |  |  |  |  |  |  |  |  |  |  |  |  |  |  |  |  |  |  |  |  |  |  |
| --- | --- | --- | --- | --- | --- | --- | --- | --- | --- | --- | --- | --- | --- | --- | --- | --- | --- | --- | --- | --- | --- | --- | --- | --- | --- | --- | --- | --- | --- | --- |
|  | **LEGEND** |  |  |  |  |  |  |  |  |  |  |  |  |  |  |  |  |  |  |  |  |  |  |  |  |  |  |  |  |  |
|  | **Fold change subset 1 / subset 2** |  |  |  |  |  |  |  |  |  |  |  |  |  |  |  |  |  |  |  |  |  |  |  |  |  |  |  |  |  |
|  |  | 0 |  |  |  |  |  |  | 1 |  |  |  |  |  | 7 |  |  |  |  |  |  |  |  |  |  |  |  |  |  |  |
|  |  |  |  |  |  |  |  |  |  |  |  |  |  |  |  |  |  |  |  |  |  |  |  |  |  |  |  |  |  |  |
|  | **Table 2a** |  |  |  |  |  |  |  |  |  |  |  |  |  |  |  |  |  |  |  |  |  |  |  |  |  |  |  |  |  |
|  | **Differential expression tumor** | CTLA-4 | | PD-1 | | PD-L1 | | PD-L2 | | VEGFR1 | | VEGFR2 | | VEGFR3 | | HGF | | MAGEA3 | | MUC1 | | MET | | HER2 | | KIT | | EGFR | |  |
|  | Adenous vs. Squamous | 0,90 | | 1,17 | | 0,78 | | 0,98 | | 0,74 | | 0,75 | | 0,62 | | 1,03 | | 0,39 | | 1,42 | | 0,90 | | 0,89 | | 1,10 | | 0,97 | |  |
|  | Ex-smoking vs. Active smoking | 0,90 | | 0,77 | | 4,16 | | 1,45 | | 0,87 | | 0,89 | | 0,55 | | 0,61 | | 4,89 | | 0,93 | | 1,13 | | 1,33 | | 0,39 | | 0,56 | |  |
|  | CRP low vs. CRP high | 0,76 | | 0,82 | | 0,43 | | 0,76 | | 1,25 | | 1,77 | | 1,24 | | 0,92 | | 0,28 | | 6,21 | | 1,29 | | 1,86 | | 5,62 | | 0,45 | |  |
|  | no COPD vs. COPD | 1,65 | | 1,10 | | 0,93 | | 0,89 | | 0,78 | | 0,79 | | 1,26 | | 1,14 | | ∞ | | 0,95 | | 0,68 | | 0,85 | | 0,93 | | 0,87 | |  |
|  | Male vs. Female | 1,12 | | 0,75 | | 2,77 | | 1,18 | | 1,84 | | 0,69 | | 2,99 | | 1,52 | | 2,17 | | 0,41 | | 1,52 | | 0,79 | | 0,53 | | 1,65 | |  |
|  | <70 years vs. ≥70 years | 1,06 | | 1,06 | | 0,58 | | 1,23 | | 0,57 | | 1,10 | | 0,53 | | 1,45 | | 1,39 | | 2,17 | | 0,33 | | 1,11 | | 0,56 | | 0,46 | |  |
|  |  |  | |  | |  | |  | |  | |  | |  | |  | |  | |  | |  | |  | |  | |  | |  |
|  | **Table 2b** |  | |  | |  | |  | |  | |  | |  | |  | |  | |  | |  | |  | |  | |  | |  |
|  | **Differential expression lung** | CTLA-4 | | PD-1 | | PD-L1 | | PD-L2 | | VEGFR1 | | VEGFR2 | | VEGFR3 | | HGF | | MAGEA3 | | MUC1 | | MET | | HER2 | | KIT | | EGFR | |  |
|  | Adenous vs. Squamous | 0,90 | | 1,17 | | 0,78 | | 0,98 | | 0,74 | | 0,75 | | 0,62 | | 1,03 | | 0,39 | | 1,42 | | 0,90 | | 0,89 | | 1,10 | | 0,97 | |  |
|  | Ex-smoking vs. Active smoking | 0,91 | | 0,93 | | 1,00 | | 0,81 | | 1,09 | | 0,97 | | 0,88 | | 1,09 | | ∞ | | 0,90 | | 1,23 | | 1,12 | | 0,79 | | 0,93 | |  |
|  | CRP low vs. CRP high | 0,88 | | 0,83 | | 0,64 | | 0,87 | | 0,72 | | 0,78 | | 0,95 | | 1,03 | | 0,42 | | 1,16 | | 0,74 | | 0,91 | | 0,94 | | 0,90 | |  |
|  | no COPD vs. COPD | 1,65 | | 1,10 | | 0,93 | | 0,89 | | 0,78 | | 0,79 | | 1,26 | | 1,14 | | ∞ | | 0,95 | | 0,68 | | 0,85 | | 0,93 | | 0,87 | |  |
|  | Male vs. Female | 1,38 | | 0,87 | | 1,53 | | 0,87 | | 1,12 | | 1,08 | | 1,38 | | 1,01 | | 0,63 | | 1,00 | | 1,32 | | 1,08 | | 0,74 | | 1,09 | |  |
|  | <70 years vs. ≥70 years | 0,75 | | 0,78 | | 0,44 | | 0,78 | | 0,91 | | 1,25 | | 1,45 | | 1,31 | | ∞ | | 0,66 | | 0,63 | | 0,85 | | 1,44 | | 0,80 | |  |
|  |  |  |  |  |  |  |  |  |  |  |  |  |  |  |  |  |  |  |  |  |  |  |  |  |  |  |  |  |  |  |

|  |  |  |  |  |  |  |  |  |  |  |  |  |  |  |  |  |  |  |  |  |  |  |  |  |  |  |  |  |  |  |
| --- | --- | --- | --- | --- | --- | --- | --- | --- | --- | --- | --- | --- | --- | --- | --- | --- | --- | --- | --- | --- | --- | --- | --- | --- | --- | --- | --- | --- | --- | --- |
|  | **Table 2a** |  |  |  |  |  |  | **Validation set** | | | | | | | | | | | | | |  |  |  |  |  |  |  |  |  |
|  | **Differential expression tumor** | CTLA-4 | | PD-1 | | PD-L1 | | PD-L2 | | VEGFR1 | | VEGFR2 | | VEGFR3 | | HGF | | MAGEA3 | | MUC1 | | MET | | HER2 | | KIT | | EGFR | |  |
|  | Adenous vs. Squamous | 1,174 | | 1,176 | | 0,723 | | 0,904 | | 1,351 | | 3,031 | | 1,574 | | 1,838 | | 0,438 | | 5,938 | | 2,24 | | 0,499 | | 2,42 | | 1,928 | |  |
|  | Ex-smoking vs. Active smoking | 0,736 | | 0,767 | | 0,865 | | 0,799 | | 0,972 | | 1,239 | | 1,075 | | 1,202 | | 0,886 | | 1,286 | | 0,829 | | 1,069 | | 0,865 | | 0,826 | |  |
|  | CRP low vs. CRP high |  | |  | |  | |  | |  | |  | |  | |  | |  | |  | |  | |  | |  | |  | |  |
|  | no COPD vs. COPD | 1,248 | | 1,158 | | 0,697 | | 1,113 | | 1,333 | | 1,707 | | 1,608 | | 1,652 | | 0,674 | | 1,385 | | 1,285 | | 1,088 | | 0,794 | | 1,442 | |  |
|  | Male vs. Female | 0,814 | | 0,835 | | 1,08 | | 1,01 | | 0,941 | | 0,593 | | 0,828 | | 0,791 | | 1,542 | | 0,677 | | 0,895 | | 1,293 | | 0,835 | | 0,87 | |  |
|  | <70 years vs. ≥70 years | 1,041 | | 0,995 | | 0,975 | | 0,981 | | 1,057 | | 1,07 | | 1,006 | | 1,161 | | 0,939 | | 0,984 | | 1,156 | | 0,876 | | 1,12 | | 0,811 | |  |
|  |  |  | |  | |  | |  | |  | |  | |  | |  | |  | |  | |  | |  | |  | |  | |  |
|  | **Table 2b** |  | |  | |  | | **Validation set** | | | | | | | | | | | | | |  | |  | |  | |  | |  |
|  | **Differential expression lung** | CTLA-4 | | PD-1 | | PD-L1 | | PD-L2 | | VEGFR1 | | VEGFR2 | | VEGFR3 | | HGF | | MAGEA3 | | MUC1 | | MET | | HER2 | | KIT | | EGFR | |  |
|  | Adenous vs. Squamous |  | |  | |  | |  | |  | |  | |  | |  | |  | |  | |  | |  | |  | |  | |  |
|  | Ex-smoking vs. Active smoking | 1,246 | | 0,967 | | 1,299 | | 1,38 | | 1,335 | | 1,238 | | 1,373 | | 1,128 | | 1,322 | | 0,687 | | 0,782 | | 1,008 | | 0,991 | | 0,934 | |  |
|  | CRP low vs. CRP high |  | |  | |  | |  | |  | |  | |  | |  | |  | |  | |  | |  | |  | |  | |  |
|  | no COPD vs. COPD | 3,578 | | 2,191 | | 1,134 | | 0,848 | | 1,558 | | 1,182 | | 1,106 | | 1,251 | | 0,478 | | 1,11 | | 1,288 | | 1,632 | | 1,163 | | 1,225 | |  |
|  | Male vs. Female | 1,035 | | 0,946 | | 0,952 | | 0,942 | | 0,988 | | 0,945 | | 0,976 | | 1,154 | | 2,606 | | 1,181 | | 0,948 | | 0,967 | | 0,886 | | 0,933 | |  |
|  | <70 years vs. ≥70 years | 0,871 | | 0,797 | | 1,05 | | 1,155 | | 0,897 | | 0,881 | | 0,942 | | 0,99 | | 1,27 | | 1,014 | | 1,013 | | 0,903 | | 1,218 | | 0,866 | |  |
|  |  |  |  |  |  |  |  |  |  |  |  |  |  |  |  |  |  |  |  |  |  |  |  |  |  |  |  |  |  |  |
